# Practical Medicine

**Published:** 1878-01

**Authors:** 


### I. Practical Medicine.

Intra-uterine Vaccination.—Bollinger (*Allg. Wiener Med. Zeitung*). In a paper on variola and vaccina (in Volkmann's series of clinical lectures), Prof. B. mentions a fact hitherto little known, but well confirmed by Reiter's excellent experiments, viz., that a vaccine virus, even in the dilution of one part to 1600, retains its specific efficiency, provided it is offered a large surface to act upon (such as that created by a vesicant). He also affirms that the blood is the agent through which all parts of the body are infected with the virus. From these facts the lecturer infers that the fœtus, which has been impregnated in utero with the virus, will possess immunity against vaccine, or variolous virus, for some time after birth. He therefore urges the re-vaccination of women in the eighth month of pregnancy. In cases where this re-vaccination is successful, the infants would be protected from their birth against one of the most dangerous of diseases. And where the re-vaccination was not successful, B. considers it worth trying to render the fœtus in utero secure, by subcutaneous or intra-venous vaccination of the mother.

Hereditary Migraine of Thirty Years' Standing Cured by Static Electricity.—Dr. Hache. (*L'Union Médicale*, Oct. 4, 1877.) M. X——, born of a gouty father and dyspeptic mother, suffered during childhood from great gastro-intestinal irritability. Thanks, however, to good management, he acquired robust health and a good constitution. But from early childhood he had been affected with headaches, which have been the torment of his life.

The attacks, extremely painful, have nearly always been connected with digestive troubles, accompanied with vomiting of food or bilious matter, and lasted from twelve hours to two days. They returned almost every week, under very diverse influences, change of diet, or of the hour of eating, cold, moral emotions, etc., etc. Nevertheless, repose of mind, residence in the country or by the seaside, travels, etc., diminished the attacks, and prevented their occurrence for some weeks.

Such was the situation of M. X—— until the age of 45 years. Engaged in important financial business, which demanded assiduous labor, and entailed great responsibility, he has been obliged to bring to bear great moral energy to overcome his neuralgic attacks, when he could not give up business. Happily, vigorous exercise, fencing, the chase, and horsemanship, which have occupied his leisure hours, have developed his muscular vigor, and neutralized the neuralgic predominance.

In 1867, to the habitual sufferings, were added a vague rheumatism, frequent furuncular eruptions, and an obstinate granular pharyngitis. The waters of Aix-les-bains were advised, with benefit to the secondary troubles, but the migraine remained unaffected. It was even complicated during two years with an acid gastralgia, rebellious to all calmatives, requiring the constant use of gaseous alkaline waters and charcoal, with an exclusively animal diet.

In October, 1875, Dr. Arthuis was consulted. After diminishing the gastric symptoms with wine of pepsine, he recommended, for the general treatment, the use of static electricity, which has been his special object of study for eight years.

M. X—— received twenty-five daily applications of fifteen minutes each; then for three weeks, every two days; and finally, every third day; the total number of electrizations being forty.

After the fifteenth sitting, he had a most violent attack of migraine; but this was the last. At the same time with the hemicrania, the gastric symptoms ceased gradually, and all food was well borne. Sometimes still, at long intervals, a painful point is felt, limited to a small surface of the head; but the suffering, of slight duration, is speedily moderated by topical calmatives, and is not accompanied by any trouble of the digestive functions.

Gout, hereditary in the family, appeared in M. X—— in 1871, and second time last spring, with medium intensity and without extending beyond the articulations of the foot.

It is nearly two years since treatment, and the benefit remains, although M. X—— has had an excess of business, and moral emotions have not been spared him.

The cure of the double affection may then be credited to the use of static electricity, of which Dr. Arthuis has already made so many happy applications to therapeutics. L. W. C.

Early Signs of Phthisis.—Dr. Haenisch (*Deutsch Arch. f. Klin. Med. and Annales de la Soc. de Méd. de Ganel*) has studied the movements of expansion of the chest in consumptives, by means of the stethograph, and concludes from his researches that in the normal state the expansion of the chest is equal for both sides. If both apices are diseased (catarrh of the small bronchial tubes, induration, cavities), the expansion is less. It is less on the diseased side than on the sound side; which fact may serve for diagnosis.

At the same time, according to him, there should be mentioned an important characteristic for the diagnosis of commencing tuberculosis drawn from the position of the clavicles; this has been shown by Aufrecht. In the normal state the acromial extremity of the clavicles is more elevated than the sternal end. If the acromial end is depressed, it signifies that the respiratory field on this side is narrowed.

If the acromial end is found on the same plane as the sternal end, if, moreover, there exist certain other suspicious symptoms such as anæmia, pains in the different portions of the chest, etc., an affection of the corresponding apex should be thought of. As a general rule the acromial end of the clavicle is lower on the diseased side than on the sound side, and lower also in the cousumptive than in healthy persons. L. W. C.

The Use of Digitalis in Disease of the Aortic Valves.—Fothergill. (*British Med. Journal*, Oct., 1877.) The consciousness that many diseases of the heart can be treated with benefit, has now become general. Formerly the hopelessness of affecting injured or disordered valves prevented early investigators of these diseases from making an effort at systematic treatment. Consequently, they confined themselves to palliative measures and the relief of symptoms, but the efforts which were first merely empirical guesses, have now become at once the most intelligent and complete of all our therapeutic measures. Digitalis, the most pronounced of all medicines in its action upon the heart, is now used with a clear conception of the results and mode of its action. Withering says of its use in the treatment of dropsy, "it seldom succeeds in men of great natural strength, of tense fibre, of warm skin, of florid complexion, or in those with a tight and chordy pulse; while, on the contrary, if the pulse be feeble or intermitting, the countenance pale, the lips livid, the skin cold, the swollen belly soft and fluctuating, or the anasarcous limbs readily pitting under the pressure of a finger, we may expect the diuretic effects to follow in a kindly manner." An experience of ninety years corroborates the opinion of this acute observer. Sir Henry Holland wrote (1839): "The enlarged and flaccid heart, though, on first view, it might seem the least favorable for the use of this medicine, is not so; at least we have reason to believe that in dropsical affections, so often connected with this organic change, the action of digitalis as a diuretic is peculiarly of avail." In spite of the prevalent opinion that hypertrophy was an undesirable growth of the heart, and that palpitation was due to a too powerful ventricular contraction, and therefore the heart's action should be lowered, accurate views have established themselves. This was chiefly due to the fact that digitalis, though a powerful diuretic in dropsy due to failure in the circulation, was not a diuretic when administered to healthy persons. For the relief comes through its effect upon the circulation, it stimulates the heart to more vigorous action, fills the arterial system with blood, causes a better blood pressure on the glomeruli of the kidneys, and consequently a better flow of urine. Our views, too, are modified as to palpitation and hypertrophy, for we now know that palpitation, when it is not a neurosal affection, is a laborious stroke indicating that the ventricle is overtaxed, instead of possessed of superfluous energy; nor is hypertrophy a disease *per se*, but a secondary growth to some obstruction of the blood flow, and a beneficent and compensatory change to be conserved.

Before the use of digitalis in disease of the aortic orifice is understood, we must glance at the pathology of aortic valvulitis. The function of these valves is closing the aortic orifice and preventing the regurgitation of blood into the ventricular channel on the aortic recoil. The ventricle, at each systole, throws so much blood * into the aorta, which, being elastic, is distended; the recoil of the aorta closes the aortic valves, preventing the reflux of blood which is then driven forward into the smaller arteries. When obstruction to the aortic blood flow is increased, two morbid processes ensue. First, the blood pressure is raised and the obstruction to the forward flow of blood on the ventricular systole is increased, and hypertrophy of the muscular walls follows. Thus the ventricle is enabled to cope with the increased blood flow, and can empty itself at each systole. Such is the simple hypertrophy of Bright's disease in its earlier stages; and from this cause comes most of the aortic valvulitis of advanced life; the aortic valvulitis induced by muscular effort is chiefly found in young men. We have, then, an obstruction to the blood flow, an increased blood pressure and a powerful ventricle; the result is the aorta is unduly distended at each ventricular systole. As the aortic recoil is in strict proportion to the amount of distention, the valves at the base are violently closed, which gives the accentuated aortic sound so significant of this condition. This powerful closure leads to valvulitis and a growth of connective tissue corpuscles in the valvular vela, but whether contraction of valves will follow cannot be seen. Distortion, however, is produced, and the valves either present an obstacle to the forward flow of blood, or they fail to arrest regurgitation, or these conditions may be combined. Hayden gives them as the most common of all combined valvular lesions.

When the valves become thick and stiff, and the process of chronic inflammation involves the base of each segment or cusp, and the growth of connective tissue leads to the contraction of the conus, stenosis is produced, and the ventricular wall has in its

---

* From 100 to 190 *grammes* (Herman's *Physiology*, translated by A. Gamgre. Page 62.)

contraction to overcome the resistance of stenosis of rigid valves, and of the normal blood pressure. In aortic stenosis when nature's efforts are sufficient for perfect compensation, a new equilibrium is achieved by hypertrophy of the ventricle not by retarding the ventricular contraction. So digitalis benefits, not by prolonging the systole, but by increasing the driving power until a normal blood-flow is secured. It may justly be urged that it produces contraction of the peripheral arterioles, raises the blood-pressure and creates an additional obstruction to the blood-flow ; but practically this is of no moment for it is not the blood pressure in the arteries which taxes the powers of the left ventricle but the tight stenosis against which it struggles.

In aortic regurgitation the semi-lunar valves shrivel along the free edges till they are insufficient to completely arrest the backward flow of blood. In this case hypertrophy is a means of arresting dilation rather than of increasing ventricular power; and we are too apt to assume the latter condition essential to evoke it. For hypertrophy does not always follow an obstruction to the flow forward of the blood on the ventricular systole. In anæmic systems and in chronic Bright's disease, dilatation is found instead, especially in women. It is found where there is no obstruction to the blood-flow, notably in hypertrophy of the left ventricle, so common in mitral regurgitation and in cardiac dilatation, the result of partial myocarditis accompanying pericarditis by which dilatation is limited.

When the aortic valves are rendered incomplete, the left ventricle is filled, not only by blood coming in from the auricle and pulmonary veins, but by that driven backward by the aortic rebound. The ventricle yields before this new force, and dilatation would now become marked did not hypertrophy arrest and limit it. In this connection occurs the most massive form of hypertrophy, the *cor bovina;* and we do not need the effects of digitalis, for the enlarged ventricle is already working ruin on the arterial walls both by its powerful contractions and over-distension by blood. This over-distension, which may be seen in the arteria centralis retinæ with the ophthalmoscope, produces chronic parenchymatous inflammation of the walls of the arteries and atheroma. (See article Philadelphia *Medical Times*, Aug.

7th, 1875, on Atheroma.) To administer an agent like digitalis, which, Balthazar Foster tells us, prolongs the diastole, would be injurious. What is wanted would be the antagonist of digitalis, which would lessen the diastole and limit the force of the ventricular sytole.

While this drug is harmful in the early, it is not so in the advanced stages of aortic regurgitation, for the arteries lose their elasticity so that the arterial recoil is diminished. This would be beneficial, were it not the force which feeds the nutrient vessels of the heart itself, consequently the hypertrophy of aortic regurgitation, though the most massive, is the least durable of all its forms. Mauriac gives the reason for this, and Balthazar Foster brilliantly illustrates it by showing that when an aortic valve is ruptured by violence, the duration of the hypertrophy depends on which valve is injured. If the torn valve have a coronary orifice behind it, the hypertrophy is brief, and the downward progress of the case is swift ; but if the valves, behind which the coronary arteries spring, are the injured ones, the complete valves arrest, to some extent, the backward flow, and so the integrity of the muscular wall is maintained, and the hypertrophy is more lasting. (*Clin. Med. and Times and Gazette*, December, 1873.)

When molecular decay begins to cut down hypertrophy, it is assisted by impaired tissue nutrition, faltering of the ventricle, insufficient filling of arteries, and diminished coronary flow. The muscular stricture is being undermined, and the ventricle yields at once to the dilating force of the incoming current, and rarely hesitates. Here digitalis gives relief by exciting more powerful contractions and securing better circulation, doing away with the long diastolic halt with its tendency to ventricular paresis. At this advanced stage, it may delay the inevitable end, but more cannot be expected. Its use in double aortic disease, must be determined by the nature and indications of each case, especially by the condition of the ventricular walls. When the ventrical falters, digitalis may be safely resorted to, till then it is better withheld.

We may sum up as follows :

1. Digitalis is useful in aortic stenosis. By exciting a more powerful ventricular contraction, it enables an equal bulk of blood to be driven through a narrowed orifice in an equal time, thus establishing a new equilibrium.

2. In the earlier stages of aortic regurgitation, with massive hypertrophy, it is harmful rather than useful.

3. In the later stages of aortic regurgitation, where the heart is failing from mural decay, and especially when intermitting, digitalis may be given with at least temporary advantage.

M. E. K.

THE TREMBLING IN PARKINSON'S DISEASE (paralysis agitans). —(*Progrès Medical*, Dec. 2, 1876 ; *Am. Jour. Med. Sci.*, July, 1877.) M. Charcot, in a recent lecture on paralysis agitans, particularly insists on the following points :

1. The name paralysis agitans is incorrect ; paralysis cannot be applied where muscular power is preserved. Nor is the affix agitans correct, because the trembling is absent in some well defined cases. He proposes instead, the name *Parkinson's Disease*, after the English physician.

2. He maintains that, as a rule, the head and neck do not take part in the tremor which affects the limbs and trunk, that the oscillations are communicated from the trunk. To prove this, he fastened a small stick to which a feather is attached, to the forehead of the patient. When left alone, the feather was unceasingly agitated. When the movements of the upper limbs were arrested, the feather was still.

3. He insists that tremor is not a necessary symptom of Parkinson's disease, for it may be so slight as to be imperceptible to the patient ; it may not appear for several years, or it may be entirely absent.

In some cases the stiff attitude, the extreme slowness of movements, the expression of hebetude, caused by immobility of features, the involuntary flow of saliva, and the interference with speech, have caused this affection to be mistaken for softening of the brain. Usually when this error has been made, the rigidity was especially marked on one side. The intellectual faculties, however, remain intact in Parkinson's disease.

M. E. K.

TREATMENT OF CATARRH OF THE BLADDER.—Prof. Edlefsen. (*Memorabilien XXI., No.* 6.) Injections of pure medicated water into the bladder are objected to by the professor. He only proceeds to such measures when internal remedies have failed. He agrees with Dittel in saying "that only in cases of the greatest necessity, should an instrument be passed into the bladder."

The professor recommends as a new remedy for catarrhal cystitis, chlorate of potash. The medicine, in sufficient quantity, can be introduced per os. In this way, while its curative effects on the bladder are obtained, the system is not overloaded with the drug. The professor further thinks that where it is required, chlorate of potash, in solution, could be injected into the bladder.

The unfavorable results of treatment in catarrh of the bladder, the author thinks, are owing to the treatment being based on incorrect suppositions, and to a timidity on the part of physicians in using certain active remedies. They either were renounced entirely or used at improper times. The most active remedies in this disease are bals. copaibæ and ol. terebenthinæ.

Very few cases of catarrh of the bladder, when treated from the beginning with either of these remedies, would long persist, and those cases which resist this treatment will most probably be due to some organic trouble of the bladder. The professor thinks that no remedy will so quickly cause an alkaline urine in a case of catarrh of the bladder to become acid, as turpentine and copaiba. These remedies diminish the catarrhal secretion of the bladder.

From considerable experience, the professor can conscientiously recommend the potassic chlorate. It does not interfere with the function of the stomach, is harmless, and where turpentine is contra-indicated, replaces it completely.

The mucous membrane of the bladder resembling the mucous membrane of the mouth and pharynx, the professor reasoned that he might expect the same results from the chlorate on the membrane of the bladder, as upon the membrane of mouth and pharynx, and experience proved that he was right. The effect of the remedy is not only to lessen the hyperæmia and the catarrh of the membrane, but it also hastens the healing of ulcers. Prof.

E. thinks it has a favorable influence on the growth of the epithelial coating.

His favorite prescription is: potass. chlorat., 15·0; aq. dest. 300·0. A tablespoonful every two or three hours.

The professor lays much stress upon the mode of administration. It is desirable to have a continuous action of the remedy. When the remedy can not be well borne, aq. laurocerasi is added.

